# Impact of distance monitoring service in managing healthcare demand: a case study through the lens of cocreation

**DOI:** 10.1186/s12913-022-08164-2

**Published:** 2022-06-21

**Authors:** Amia Enam, Heidi Carin Dreyer, Luitzen De Boer

**Affiliations:** 1 Department of Industrial Economics and Technology Management, Faculty of Economics and Management, Norwegian University of Science and Technology, Sentralbygg 1, 1365, Gløshaugen, Alfred Getz’ vei 3, Trondheim, Norway; 2 Department of Industrial Economics and Technology Management, Faculty of Economics and Management, Norwegian University of Science and Technology, Sentralbygg 1, Gløshaugen, Alfred Getz vei 3, Trondheim, Norway; 3 Department of Industrial Economics and Technology Management, Faculty of Economics and Management, Norwegian University of Science and Technology, Sentralbygg 1, Gløshaugen, Alfred Getz vei 3, Trondheim, Norway

**Keywords:** Telemedicine, Distance monitoring, Co-creation, Demand, Resource, Management

## Abstract

**Background:**

There is a consensus among healthcare providers, academics, and policy-makers that spiraling demand and diminishing resources are threatening the sustainability of the current healthcare system. Different telemedicine services are seen as potential solutions to the current challenges in healthcare. This paper aims to identify how distance monitoring services rendered for patients with chronic conditions can affect the escalating demand for healthcare. First, we identify how distance monitoring service changes the care delivery process using the lens of service cocreation. Next, we analyze how these changes can impact healthcare demand using the literature on demand and capacity management.

**Method:**

In this qualitative study, we explore a distance monitoring service in a primary healthcare setting in Norway. We collected primary data from nurses and general physicians using the semi-structured interview technique. We used secondary patient data collected from a study conducted to evaluate the distance monitoring project. The deductive content analysis method was used to analyze the data.

**Result:**

This study shows that the application of distance monitoring services changes the care delivery process by creating new activities, new channels for interaction, and new roles for patients, general physicians, and nurses. We define patients’ roles as proactive providers of health information, general physicians’ roles as patient selectors, and nurses’ roles as technical coordinators, data workers, and empathetic listeners. Thus, the co-creation aspect of the service becomes more prominent demonstrating potential for better management of healthcare demand. However, these changes also render the management of demand and resources more complex. To reduce the complexities, we propose three mechanisms: foreseeing and managing new roles, developing capabilities, and adopting a system-wide perspective.

**Conclusion:**

The main contribution of the paper is that it demonstrates that, although distance monitoring services have the potential to have a positive impact on healthcare demand management, in the absence of adequate managerial mechanisms, they can also adversely affect healthcare demand management. This study provides a means for practitioners to reflect upon and refine the decisions that they make regarding telemedicine deployment and resource planning for delivering care.

**Supplementary Information:**

The online version contains supplementary material available at 10.1186/s12913-022-08164-2.

## Introduction

### Background

There is a consensus among healthcare providers, academics, and policy-makers that spiraling demand and diminishing resources are threatening the sustainability of the current healthcare system [1, 2]. The rise in chronic ailments, multi-morbidities, and the aging population have increased healthcare demand, and the existing resources fall short of meeting these demands. The global burden of chronic disease is enormous, and it is currently one of the top ten causes of death worldwide [3]. Illnesses such as chronic obstructive pulmonary disease (COPD) and heart failure (HF) increase hospital admissions and the number of visits to general physicians, exerting additional pressure on healthcare resources and reducing the quality of care [4, 5]. These chronic diseases are also recognized as among the main causes of comorbidity [6] that increase the demand for healthcare even more. Telemedicine – the group of IT that facilitates the diagnosis, treatment, and monitoring of patients when distance separates the healthcare professional and the patient [7] – is seen as a potential solution for the current challenges in healthcare [8]. In general, the expectation from telemedicine implementation is that it would make the healthcare system more resource efficient [9]. Distance monitoring is one such aspect of telemedicine that is also considered to be instrumental in achieving good quality of care, particularly for chronic illnesses, such as COPD, diabetes, HF, depression, and cancers [10–12].

Several existing studies have investigated how distance monitoring service affects clinical efficacy and patient safety, which are essential to deciding whether this service is suitable for patients and to minimize the harm that might be caused by it [13, 14]. However, to implement distance monitoring service on a large scale and adopt it as a routine service, it is crucial to understand its effect on resource utilization [9, 15]. The way in which a new technology alters the existing workflow was found to be one of the main factors predicting its implementation success [16]. Therefore, it is essential to determine the changes in the care delivery process triggered by distance monitoring services. Furthermore, as previously mentioned, the global healthcare system is facing resource constraints. Hence, a telemedicine service that is not resource efficient will be much more difficult to implement and sustain, even if its clinical efficacy is well proven. Nevertheless, whether the application of distance monitoring can reduce the current discrepancies between demand and capacity in healthcare services is largely unknown [17].

Additionally, chronic ailments and multimorbidity make dynamic and diverse demands of care provision [18]. Therefore, a thorough analysis of how these demands can be managed is greatly needed [19]. Although poor management of healthcare demand and capacity has adverse effects—such as poor patient flow, reduced patient safety, communication failure, dissatisfied patients and healthcare professionals, and lost revenue—very few empirical studies have explained how the disparity between demand and capacity in healthcare can be improved [20–23].

Therefore, this paper aims to explore how distance monitoring services can affect demand and capacity management in healthcare. The objective of the paper is twofold, to identify: (1) how distance monitoring services change the care delivery process; and (2) how these changes can potentially improve the management of demand and capacity in healthcare. The guiding research questions (RQs) of the paper are as follows.

1. How do distance monitoring services change the care delivery process?
2. How do these changes in the care delivery process affect demand management in healthcare?

To answer these questions, we conducted a qualitative study of a distance monitoring service in Norway. We use co-creation of care as a theoretical lens and empirical data from a case study to answer the first RQ. The second RQ is answered through a conceptual analysis of the findings from the case study in relation to the demand and capacity management literature. The main contribution of the paper is that it demonstrates that, although distance monitoring can have a positive impact on healthcare demand management, in the absence of adequate managerial mechanisms, it can also adversely affect healthcare demand. We identify the circumstances under which distance monitoring services enable and limit the management of demand and resources in healthcare and propose enabling managerial mechanisms. This study has useful implications for both research and practice, as explained later. The remainder of the paper is structured as follows. First, we briefly discuss care co-creation and demand and capacity management strategies in healthcare leading up to the research framework; next, we explain the methodology of the study and elaborate on the case; the findings of the study are then presented, followed by the implications of the findings for research and practice; and finally, we present concluding remarks, including the opportunities for future research.

### Care co-creation

Co-creation is a much researched area in services including healthcare. This section does not provide an exhaustive review of this vast literature (e.g., [24–31]) but rather explains how this concept is used in this study. In the literature, the co-creation of service has been analyzed from two different perspectives: one proposes that service is always co-created with the customer, in our case, patients [26, 32, 33], and the other proposes that co-creation is an optional collaborative act in the customer-provider interface [34]. In summary, co-creation is about outlining and creating value through multiple repetitive collaborative processes between providers and consumers that include value proposition, resource integration, and learning processes [9]. In this study, co-creation is seen as an inherent aspect of healthcare service since patients’ participation is essential for care creation. However, we also posit that the degree of patients’ participation and collaboration between them and care providers vary from service to service. Moreover, the co-creation aspect of healthcare service can also be impacted by any change made to the service delivery process, for example, using telemedicine to provide care.

Therefore, to explore the care co-creation process of the distance monitoring service, we adopt McColl-Kennedy et al.’s (2012) dimensions of co-creation: activities, interactions, and roles [35]. Activities imply performing actions to co-create. Interactions imply engagement with others to co-create. Roles are the structures that imply individuals’ representation in the co-creation process. The central argument behind these three dimensions is that the representational practices in service affect how individuals interact with each other, in turn influencing individuals’ activities and consequently changing actors’ roles, and the process repeats itself whenever there is an occasion for change [35]. In this study, we applied the dimensions of cocreation as an enabling theory to explain the phenomenon of interest, i.e., the changes in a care delivery process [36, 37]. Using these dimensions, we explore how the application of distance monitoring changes the activities and interactions and the roles of the actors in the care creation process. We apply this framework to the service triad of patients, nurses, and general physicians (GPs) – who are the main actors of the distance monitoring service, as explained in a later section. Compared to a dyadic relationship, for example, patient-nurse or patient-GP, the service triad provides a richer context for understanding the interactions, contributions, and nature of the actors involved [38, 39].

### Demand and capacity management strategies in healthcare

The concepts of demand and capacity are more difficult to grasp, measure, and manage in the service context, including healthcare compared with that of physical goods [40]. Balancing demand and resources in healthcare is complex because services are: (1) perishable and cannot be inventoried and transported, leading to the simultaneous generation of demand and consumption of resources; (2) intangible and difficult to specify in terms of quantity and quality; and (3) characterized by a high degree of producer–consumer interaction [41–43]. In this paper, we analyze how, on a strategic level, the application of distance monitoring services can impact the management of demand and capacity. Therefore, we synthesize different demand and capacity strategies found in the service literature. We do not limit our analysis to the healthcare literature since distance monitoring can trigger a strategy that has yet not been used in healthcare services.

Demand management strategies are aimed at one or several of the following goals: (1) increase or decrease demand, (2) change the timing of the demand, and (3) rechannel demand to other resources [41, 44–46]. Focusing on different client segments for different demand periods is a demand strategy [47, 48], for example, dividing patients for walk-in visits and scheduled appointments [46] or into online and in-person visits [49]. Informing and educating customers constitute another demand management strategy that focuses on informing customers about peak and slack demand periods so they can make informed decisions about when to seek service [50, 51]. The referral system, i.e., patients requiring a referral from GPs to access specialized healthcare, is also a demand management strategy in which a high threshold is created to reduce the demand in hospitals [52].

Capacity management strategies aim to acquire and allocate resources to minimize waiting time and idle capacity while meeting demand [23, 53, 54]. In a service context, capacity is the maximum amount of output that is available in a given period with a predefined level of resources, i.e., workforce, equipment, and facilities [55]. Capacity management strategies include a flexible and multiskilled workforce, subcontracting facilities and equipment, and increasing client participation [23, 43, 47, 56, 57]. A flexible and multiskilled workforce adds more options in staff scheduling and allocation, and employees can easily shift from task to task as required [46]. Such as workforce includes the recruiting of temporary and on-call staff and awarding of overtime payments [58]. Subcontracting facilities and equipment indicates sharing capacity with other organizations, for example, using the same pathological laboratory by different clinics [41]. Examples of increasing consumer participation include restaurant buffet services and airport self-check-in [47, 50].

### Research framework

**Fig. 1:**
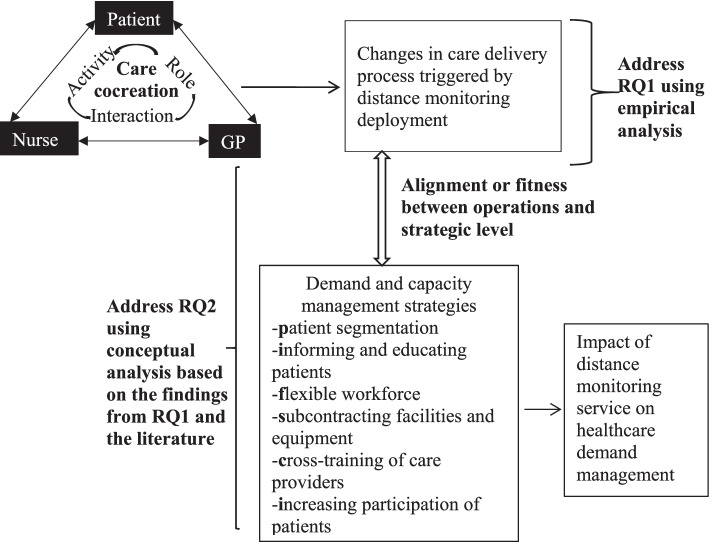
Research framework with applied theoretical approaches

Figure 1 exhibits the research framework for the study. The framework is built on the three dimensions of care creation and the demand and capacity management strategies. As the figure implies, we first identify the changes in the care delivery process triggered by distance monitoring service in terms of the activities, interactions, and roles of the patient, GP, and nurse, who are the main actors in the care delivery process. Then, we analyze how these changes at the operational level are aligned with demand and capacity management strategies to understand how distance monitoring services can contribute to improving the management of healthcare demand. A good fit between operational and strategic levels is crucial for any service to be implemented and sustained [59]. Nevertheless, technology-enabled services are often criticized for missing this fit, falling short in fulfilling the objective(s) and ending up as abandoned services [60, 61]. Therefore, we focus on the fit between the operational changes in the care delivery process and demand-capacity management strategies to argue how distance monitoring services can impact healthcare demand management. The methodology used to conduct the study is described in the next section.

## Methodology

A case study is a suitable approach to understand the complexities of a certain phenomenon in relation to the context of the phenomenon [62]. To explore and understand telemedicine services in depth, this paper chooses a case study as its research strategy, in which the unit of analysis is the care delivery process in the service triad of patients, GPs, and nurses. Starting as a trial project in 2016 by the Norwegian government, distance monitoring services are now available in six municipalities that use the same telemedicine technology to support elderly patients with chronic diseases, such as chronic obstructive pulmonary disease, diabetes, and cancer [63]. As part of a project planned and governed by the central health authority, these municipalities follow the same service configuration. Initially, four municipalities agreed to contribute to our study. However, due to the COVID-19 pandemic, two could not participate since their limited human resources had to focus on the pandemic. Therefore, we use data collected from two municipalities to typify the distance monitoring service in this paper.

### Telecare and traditional care

In Norway, healthcare is fundamentally a public service. It has two parts: (1) primary care, consisting of GPs and municipality care; and (2) specialized care, consisting of hospitals providing diagnosis-specific services. Residents in Norway are assigned to GPs, who are the gatekeepers to specialized care—i.e., they refer 16 to hospitals if needed. Municipalities focus on keeping residents functional and healthy for as long as possible to minimize demands on home care and nursing homes. Thus, traditional care service in Norway consists of GPs, specialized hospitals, emergency care by hospitals and municipalities, and social and homecare by the municipality.

**Fig. 2:**
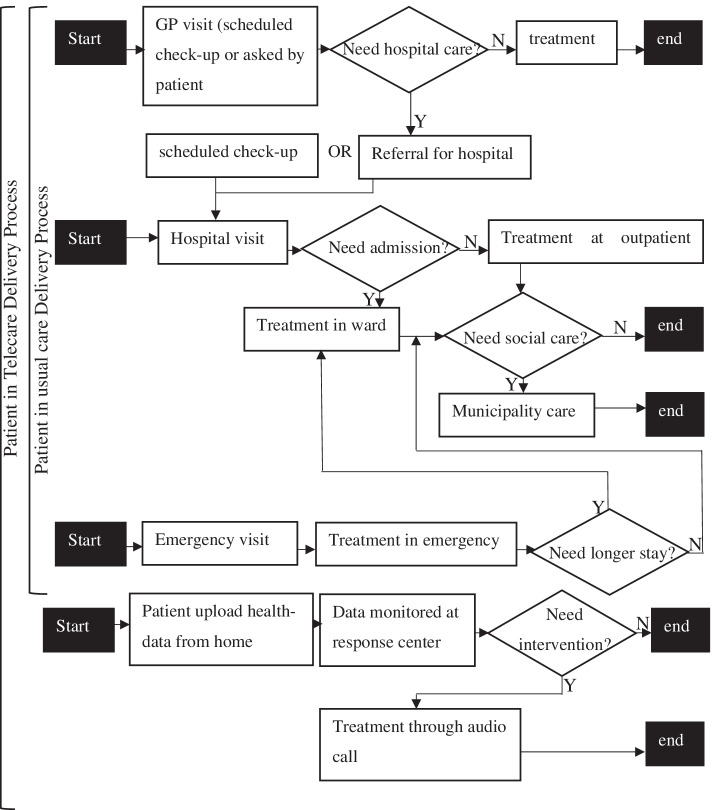
Telecare and traditional care delivery process

The main objectives of the distance monitoring service (hereinafter, telecare) are to: (1) provide patients with better care and opportunities to control their illnesses better; and (2) reduce resource utilization across primary and specialized healthcare [64]. It is noteworthy that patients enrolled in telecare have equal access to their GPs and hospitals as patients who are not enrolled in telecare but receive traditional care only. Traditional care and telecare services contain three and four types of care episodes, respectively (Fig. 2), i.e. series of healthcare-related events that a patient undergoes [65].

### Ethical consideration

This study was approved by the Norwegian Centre for Research Data (NSD), and the reference number of the application is 800636. The committee assessed the application and decided that “the processing of personal data in this project will comply with data protection legislation, presupposing that it is carried out in accordance with the information given in the Notification Form and attachments, dated 08.10.2019. Everything is in place for the processing to begin.” We also sought permission (reference no. 58059) from the regional committee for medical and health research ethics (REK) to interview patients. However, the committee assessed that the project falls outside the scope of the health research act (ACT 2008–06-20 no. 44) and therefore could be conducted without REK’s approval. Following the NSD guidelines, written consent was obtained for each interview, and the data were anonymized and stored on the researcher server of the university at which the project was conducted.

### Data collection and analysis

We conducted the exploration of the literature, data collection, and analysis in parallel and iteratively from September 2019 to January 2021. This study uses qualitative data collected from multiple sources using multiple methods to identify whether these data corroborate each other and to build a coherent narrative for the case. This triangulation of data strengthens the findings and increases the study’s quality [62, 66]. A brief description of data collection follows.

1. Non-participatory workshop observation

To enhance contextual insight into telecare services, the first author attended a workshop as a non-participatory observer [67] in the initial phase of the study (December 2019). This workshop, at which 12 GPs and nine nurses from all six municipalities shared their telecare experiences, was arranged by the Norwegian health ministry. This workshop helped us to understand the configuration of the telecare service followed by all of the municipalities and allowed us to contact these municipalities for in-depth data collection.

2. Documents and archives

We read and analyzed several project reports, which provided specific and precise information about telecare, helping us to understand its initiation and the evolution of the project. While we read many documents and newspaper articles, six telecare evaluation reports published from 2018 to 2021 were thoroughly read.

3. Semi-structured interviews

We developed an interview protocol [62] after a preliminary review of the literature and participation in the telecare service workshop. The interview protocol comprised different sets of questions for different respondents, i.e., nurses, management, GPs, the app developer, and patients. The aim was to gather in-depth information to build a comprehensive narrative capturing the changes in the care delivery process and their effects on resource utilization. To recruit the respondents, an invitation letter was sent to the management of the municipality care services, and one manager from each municipality became our main point of contact (POC). With the help of the POC, we sent invitation letters to all groups of respondents. The invitation letter provided an overview of the study project, including its objectives and context and the time required from the respondents. We did not provide the interview questions beforehand since we followed a semi-structured interview technique [68]. The inclusion criterion for participating in the study was that the respondent needed to have at least 3 months of experience with the distance monitoring service. We did not consider any demographic characteristics, such as age or gender, as criteria for recruiting respondents since the focus was to collect information about the care delivery process as a comprehensive system. To determine the number of respondents for all groups except app developer, we relied on data saturation – the stage of data collection in which information received from the respondents becomes repetitive and the likelihood of obtaining new information becomes negligible [69]. As Table 1 shows, we interviewed only one respondent in relation to app development since the other personnel directly connected to this project no longer worked for this software company. However, the app developer interviewed by us is the project leader and had comprehensive knowledge about the development and deployment of the distance monitoring app. Moreover, the information collected from this respondent was corroborated by information collected from other sources. The duration of the interviews varied from 45 to 75 min. After transcribing the interviews verbatim, we conducted follow-up interviews with four respondents (1 nurse, 1 GP, and 2 managers) to clarify issues raised in previous discussions. All of the respondents were asked on the consent form whether they would like to receive and check the transcript or audio of the interview, and none have made such requests. Table 1 provides an overview of the interview respondents. Only one patient was interviewed before the pandemic started; due to restrictions, face-to-face meetings became infeasible, and digital meetings with patients were not approved ethically. Therefore, we used secondary data on the patients’ feedback.

**Table 1:** Overview of interview respondents

Respondents		Number of interviewees	Number of interviews
Nurses	Monitoring nurse	8	8
	Coordinating nurse	2	3
Management	Manager in the municipality care	4	6
	Human resource manager in response center	2	2
General physicians (GP)		3	4
Distance monitoring app developer		1	1
Patients		1	1
Total		21	25

4. Secondary patient data

Despite having little to no control over data quality, using secondary data sources is a valid and even recommended research strategy, especially when primary data are unavailable [68]. Since the patient’s voice is significant for understanding the co-creation of care, we decided to use secondary data when patient interviews were no longer an option. To include patients’ voices, we used official reports that are accessible to the public and are considered a valid source of secondary data (e.g., [70]). We used the assessment report of the project [71], consisting of 15 patients’ interviews and one group discussion. The report aimed to understand patients’ perception of telecare, in agreement with our aim of interviewing the patients. We undertook several precautions to ensure the quality of the data: (1) to minimize researchers’ bias, direct patient feedback was used, instead of the report’s analysis; (2) the data were collected in late 2019 and early 2020, overlapping with our data collection period; and (3) one of the authors of the report, a nurse, was interviewed to understand the context of the data collection and the types of questions asked.

Following a systematic combining approach [72], we started analyzing the data in the early phase of data collection. Initially, collected data were used to edit the interview protocol and identify literature that supported or opposed our findings [66]. From emerging data and an iterative literature review, we developed a guiding research framework (Fig. 1). Eventually, a case diary was developed, including transcriptions of all interviews, workshop notes, and information extracted from project reports. It should be mentioned that, whereas data collected from management and app developer: (1) enriched our understanding of the context, which is essential for case studies [62]; and (2) were used for data triangulation [66]; the data collected from nurses, patients, and GPs were further analyzed.

We applied the content analysis method [73] to analyze the data. Between deductive and inductive content analyses, we chose deductive analysis since we had developed the research framework and identified the constructs for analyzing the data. Therefore, the dimensions of co-creation—activities, interactions, and roles—were used to categorize the data. To extract relevant information for these dimensions, we used the following predefined criteria: (1) tasks; (2) actors; (3) the link between different tasks and the actors; and (4) the different tools used, e.g., telemedicine app and different EHRs. The next task was to arrange these activities and interactions chronologically to visualize the care co-creation process. Using a visual mapping technique [74], the complex process of care co-creation was outlined using pen and paper, a schematic version of which is exhibited in Fig. 2. The different actors’ roles became visible through this exercise.

We continuously collaborated, both externally with the respondents and internally among co-authors throughout the analysis process. The analysis of the data was conducted in close collaboration with all of the authors. The first author transcribed the interviews and developed the case diary, which was thoroughly reviewed by the other two authors. During this process, the authors identified some gaps in the narration that were closed by conducting four follow-up interviews as mentioned earlier. Moreover, several statements made by the first author were questioned by the other two, and upon further discussion and analysis of the interview transcriptions, the statements were adjusted. We followed a similar process during the grouping and plotting of the data in our framework. Moreover, the findings were presented to one of the GPs and one management representative to identify whether our analyses were based on any wrong assumptions.

## Results

### Changes in the care delivery process

This section answers the first RQ: How does distance monitoring service change the care delivery process? Using co-creation as the theoretical lens, we describe how the application of a distance monitoring service changes the activities, interactions, and roles of the main actors in the care delivery process, i.e., patients, GPs, and nurses, and finally, we summarize the findings at the end of the section.

### Patients

Patients in telecare are required to report their health condition regularly to the response center using a distance monitoring application (app) on a mobile phone or tablet. First, patients measured various health parameters, such as blood pressure and oxygen levels, using different equipment. Patients found this exercise helpful.

“It is good to do and see my own health measures, and thus I don’t have to wonder about how my health is.” - Patient 3

“You can measure your blood pressure if you are feeling strange, and that’s great!” - Patient 4

Moreover, the app asks questions about factors such as the color of the cough and breathing condition. To answer these questions, the patients must systematically think about and assess their health.

“I believe I can feel my health better. I observe the symptoms (worsening signs) much earlier.” - Patient 1

The app itself conducts a quick data assessment and provides the patient with a simple result regarding their health status in terms of the green, yellow, or red zone, ranging from good to bad. Furthermore, patients can write messages in the app to communicate with the response center, as one nurse explained.

“His health assessment is always on the yellow side, but he sends us a message saying, ‘I am okay; don’t call me.” – Nurse 6

Real-time data sharing and interaction with nurses can cause the patient to feel safer at home, as one patient explained, “The fact that someone is watching out for you makes this service very helpful.” Patients, however, can find it inconvenient to perform these activities regularly, especially when they are not feeling sick.

“I don’t feel like thinking about my disease every day…” – Patient 2

“You may not want to think about your disease on the days you are fine.” – Patient 3.

While patients’ perceptions about telecare service vary, one important aspect that has emerged is that, for this service to function, patients’ participation in the care co-creation process must be increased. Measuring and providing health data are new activities added to patients in telecare services, and the distance monitoring app has become the new channel of interaction between patients and nurses. Thus, patients play an increasingly important role in the care delivery process. We define this new patient role as a proactive supplier of health data.

### General physicians (GPs)

In the telecare service, GPs are responsible not only for holding consultations and providing referrals but also for recruiting patients for this service. The recruitment process consists of the following activities: (1) identifying suitable patients for telecare depending on patients’ health status and ability to use the app; (2) introducing and offering telecare services to suitable patients; (3) providing coordinating nurses with lists of patients willing to join; and (4) devising treatment guidelines for individual patients included in telecare. This individual treatment plan is the basis for the treatment-related decisions made by monitoring nurses in the response center. Thus, GPs in the telecare service interact with these nurses, either in person or via telephone and e-messages.

GPs believe that the patient recruitment process is one of the most important aspects of telecare service and is directly connected to patient safety.

“[The] most important thing is how we include patients and which ones are to be excluded [from telecare].” – GP 1

“You do not want to include someone who will not benefit from such a service option” – GP 2

Furthermore, compared with doctors from specialized care or nurses, GPs have the most comprehensive knowledge about patients’ health and lifestyles, making them more eligible to recruit patients:

“We do not deal with one disease or diagnosis but the whole person.” – GP 3

“For some patients, I even know their grandparents, so I have a better overview of the patients to see if they fit [the telecare service].” – GP 2

Thus, we identify GPs taking a new role in recruiting suitable patients for telecare, and we define this role as a patient selector.

### Nurses

The nurses can be divided into monitoring nurses performing activities in the response center and coordinating nurses performing activities coordinating with GPs, patients, and monitoring nurses.

The coordinating nurse makes the first contact with a patient after recruitment. Next, they receive the treatment plan from the respective GP and set the app accordingly. Then, they meet the patient face to face to train him or her on using the app and other equipment. In addition to coordinating the recruitment process, the coordinating nurse also makes monthly contact with those patients not providing health data as planned. This interaction aims to establish why the patient is not cooperating and whether they can start using the app again. Last, the coordinating nurse resolves technical issues that arise at the patients’ end regarding the app and equipment. We identify this role as a technical coordinator since the coordinating nurse is responsible for technical coordination, in addition to care coordination. The following quotes from coordinating nurses exemplify this role.

“I go through the [patient] log and call those patients who are not using the app one by one. Sometimes they have some technical problems, such as passwords being erased for system updates or a lost internet connection in the tab. Then, I help to solve those.” – coordinating nurse 2

“It is easier to fix the technical issues, but it takes a lot of my time to find the patients and call them one by one to know why they are not using [the app].” – coordinating nurse 1

The monitoring nurses work in the response center and interact with patients and occasionally with GPs. First, app alerts inform them about patients’ activities. If the data are in the yellow or red zone or the patient has asked to be contacted, the monitoring nurse calls the patient. During these calls, the nurse can change the medicine or doses of an ongoing medicine according to the treatment plan. These decisions depend on how nurses interpret the data and compare them with previous data and the treatment plan. Thus, instead of seeing the patient and examining him or her, these nurses navigate through different IT systems, making sense of data and making decisions accordingly:

“To work in [a] response center, one needs to love technology.” – Monitoring nurse 8.

“It’s different here [in the response center] than my work at the hospital. Now I work a lot with [IT] systems.” – Monitoring nurse 3

“We have to be very good at handling data now and have to switch fast from one system to the other.” – Monitoring nurse 7

We define this role as data worker. Next, our data reveal that the interactions with patients also soothe them, alleviating their stress or anxiety:

“Talking to [the patient] over the phone can calm them down. Sometimes they just want to hear from you that everything is fine.” – Monitoring nurse 2

“A lot of these patients are old and live most of the day by themselves. Just calling them and asking, ‘How are you doing today?’ makes a difference for them.” – Monitoring nurse 4

“They become relaxed and end up sharing lots of things, like what to buy for their son’s birthday, or ‘My pet has not eaten today.’” – Monitoring nurse 5

We define this role as empathetic listener because it is quite different to interact with patients by telephone to understand their needs and communicate accordingly to calm them and cause them to feel better compared with having a face-to-face conversation. As one nurse put it:

“One must develop an ear [to understand patients’ needs].” – Monitoring nurse 6

Table 2 provides a summary of the identified activities and interactions in the telecare delivery process and the new roles that emerged.

**Table 2:** Changes in the care delivery process in telecare service

**Actors**	Activities	Interactions	**Identified novel roles**
Patient	Uses the equipment and app to measure and record health data Answers questions in the app Reviews the results of health assessment using the app	Meets the coordinating nurse to learn how to use the app and equipment Sends messages to nurses via the app Talks to nurses via telephone	**Proactive supplier**
General physician	Identifies and recruits suitable patients for telecare Makes first contact between the coordinating nurse and patient Develops treatment plans for individual patients	Has face-to-face interactions with patients and coordinating nurses during the recruitment process Is contacted by monitoring nurses via e-message	**Patient selector**
Municipality nurse	Coordinates the recruitment process and the patient training Tracks patients not conforming to the telecare treatment Resolves technical issues Monitors data coming from the patients in real time Interprets data to make treatment decisions based on the individual treatment plan	Calls the patients to make changes in treatment and pacify him or her Sends e-messages to GPs, either to provide information or to receive further information about particular patients Ensures that treatment plans are standard documents of communication between nurses and GPs	**Technical coordinator** **Data worker empathetic listener**

### Effect of telecare service on healthcare demand management

This section answers the second research question: How do these changes in the care delivery process affect demand management in healthcare? We analyze how the changes in the care delivery process are aligned with different demand and capacity management strategies. In this conceptual analysis, we consider the new roles of patients, nurses, and GPs as the units of change in the care delivery process because these roles simultaneously represent the changes in activities and interactions. We consider each actor and assess the extent to which the changing role(s) are aligned with demand and capacity management strategies and can potentially improve the management of healthcare demand.

### Patient as a proactive supplier: increasing client participation

Increased patient participation is a necessary condition for telecare service since only the health data and the queries uploaded in the app by the patients can initiate the service. The effectiveness of this service also depends upon on how timely a basis these data are sent. If the patients wait until they are severely sick, then a visit to the emergency or the GP is inevitable, defying the objective of telecare service. If the patients are both skilled and willing to contribute to data measuring and sharing, it is reasonable to expect reduced demand for GPs, hospital visits, and emergency admissions. Thus, ideally, increasing patients’ participation can indeed improve resource utilization, concurring with the literature [43, 46]. However, when the patients are unwilling or not sufficiently skilled to measure and register their health data regularly, the nurses cannot support the patients. The complexity of this aspect is at least twofold, as emerged in our case. First, not all patients are equally interested in helping to manage their disease, nor are they all equally skilled, as the coordinating nurses explained:

“Sometimes they forget the password or give the wrong password too many times, so the app stops working.” – coordinating nurse 1

“There is a tendency to stop using the app when a person is stable for some time.” – coordinating nurse 2.

In the current telecare service, the only mechanism focusing on managing patients’ involvement is that of the coordinating nurses, by which they contact the patients who are not conforming with their treatment plan. This mechanism does not ensure patients’ future participation, and it could become infeasible to check on individual patients manually if the number of such patients increases significantly. The high level of dependency that telecare service has on its patients, thus adds complexities in maintaining service quality. If the patients do not cooperate in supplying the data through the app, the response center cannot provide them with the timely, required support. Consequently, patients could end up visiting GPs or hospitals at the same rate as patients from traditional care, jeopardizing the effectiveness of telecare services.

### GPs’ role as patient selectors: segmenting and rechanneling clients

GPs’ role as patient selectors implies that telecare services are suitable only for patients with certain abilities and willingness and, most importantly, comparatively stable health conditions. The underlying objective of this patient segmentation is to reduce the demand for traditional care and rechannel them to the telecare service. However, for the segmentation and rechanneling strategy to function effectively, it must be ensured that telecare patients use fewer resources than patients in traditional service. Telecare service is, however, an additional channel, indicating that the patients enrolled for this service have equal access to GPs and hospitals as the patients from the traditional service. Therefore, if patients’ need for GPs and hospitals is not met or reduced by telecare, they will end up utilizing resources from both telecare and traditional services, aggravating demand escalation. One recent financial evaluation of the telecare project reported such a negative gain in resource utilization [63]. While sufficient empirical evidence is unavailable to explain this increased use of resources, the aforementioned scenario can conceptually explain the reason.

Thus, segmenting and rechanneling clients to telecare does not constitute an infallible strategy to reduce healthcare demand. GPs’ role as patient selectors could be an effective mechanism to ensure that telecare service is offered only to those patients who will benefit from it so much that their visits to GPs and hospitals will eventually be minimized. However, the absence of guidelines for patient selection in the current service configuration threatens the effectiveness of GPs’ role as patient selectors.

### Multiple roles of monitoring nurses: flexible and multiskilled workforce

A flexible and multiskilled workforce is often mentioned as an effective strategy for managing demand for services [22]. The emergence of multiple new roles for nurses in telecare services indicates the need for nurses to become flexible and be simultaneously skilled in several dimensions. The response center provides a lower threshold for patients in making real-time contact with healthcare personnel. By performing a variety of tasks, including providing technical support, interpreting data, pacifying patients, and altering treatment, nurses also add flexibility to the range of services offered through telecare. From the patient’s perspective, it is much quicker to reach and receive relevant support from nurses compared with the time necessary to consult with traditional care service. However, in the current telecare service, the training of the nurses for these novel roles is not quite visible. According to HR managers and nurses, the nurses receive on-the-job training in their initial days of employment. This training focuses on using the IT systems and app and is conducted by one or two on-duty senior nurses. No education or training on providing distant care or data interpretation is provided.

Overall, the analysis indicates that the changes in the care delivery process enable opportunities to implement several demand and capacity management strategies. However, in the current telecare service, these strategies are neither consciously devised nor managed and are devoid of proper mechanisms that could make them effective in managing healthcare demand. Consequently, the current telecare provision might not make a positive contribution to addressing the issues of spiraling demand and diminishing resources in the current healthcare system. More importantly, in the absence of proper strategic implementation, the changes in telecare services could even complicate resource distribution and lead to increased use of resources. Table 3 provides an overview of which demand and capacity management strategies are aligned with identified changes in the care delivery process and the implications for telecare service.

**Table 3:** Identified demand and capacity management strategies in the telecare service

Novel roles in the telecare service triad	Potential demand and capacity management strategies	Implications for the telecare service
Patients as proactive suppliers	Increasing clients’ participation	**Current status:** Add complexity to telecare services since the quality of patients’ contributions cannot be forecasted and controlled **Potential:** If the patients are adequately skilled and comply with care plans, the demand for GPs and specialized care would be reduced. Such patients would become more active in the care cocreation process. Consequently, they would have a better understanding of and greater control over their health status
GPs as patient selectors	Rechanneling demand	**Current status:** To ensure meaningful contributions by patients in the care cocreation process, selecting patients with the right skills and adequate willingness is essential. Otherwise, patients could end up using resources in both telecare and traditional care channels, with the possibility of increased resource utilization **Potential:** A structured guideline based on empirical evidence of clinical efficacy could ensure that the patients chosen for telecare are able to exploit the services provided by nurses and thus will require fewer GP and hospital visits
	Client segmentation	**Current status:** Individual GPs have their own way of selecting patients for the telecare service, which does not ensure effective use of telecare **Potential:** Can be a useful mechanism for: (1) categorizing the patients for telecare and traditional services based on a standardized guideline; (2) choosing the right patients for telecare so that they have a reduced need for traditional care
Nurses as technical coordinators, data workers, and empathetic listeners	Multiskilled and flexible workforce	**Current status:** In addition to tending to patients, nurses also become more active in the cocreation process in telecare. Multiple roles played by nurses render telecare responsive and easily accessible. However, the need for new competencies related to technology and distant care is not recognized; thus, adequate training is lacking, which can lead to reduced quality of care **Potential:** Nurses’ roles are essential in rendering the treatment and support of telecare effective. However, (1) formalization and systematic evaluation of the competencies needed and (2) corresponding training are essential to make these roles, and thereby the telecare service, effective

## Discussion

### Key findings

The findings of the study are twofold. First, it identifies the changes in the care delivery process of a distance monitoring service using the lens of service co-creation and the service triad. Several new activities (Table 2) are added to each of the actors, and patients’ participation in creating care becomes more prominent than that with the traditional service. The distance monitoring app becomes the new channel of interaction between patients and nurses, whereas the interactions between GPs and nurses become more frequent. Several new roles emerge for patients, GPs, and nurses, which we define as proactive suppliers of health data, patient selectors, technical coordinators, data workers, and empathetic listeners, respectively.

Second, this study indicates that distance monitoring service, on the operational level, creates several opportunities for better management of healthcare demand since the changes in the care delivery process fit with four demand-capacity management strategies found in the literature, namely increasing patients’ participation, segmenting and rechanneling the patients, and flexible and multiskilled nurses. This finding is in line with the findings of studies focusing on preventive and self-management care for patients with chronic ailments [5, 75]. However, on the strategic level, these strategies have yet to be devised and implemented. We demonstrate (current statuses in Table 3) that, in the absence of adequate support from the strategic level, the changes identified in the care delivery process have limited and, to an extent, adverse effects on demand management. Our analysis additionally indicates that the care delivery process becomes increasingly dependent on patients and requires new skills for nurses and GPs. These changes indicate that telecare provides provisions for co-creating care by involving different actors, most importantly the patient, in the care delivery process. These changes simultaneously add complexities to the care delivery process, which can hamper the quality of care and resource efficiency, if not addressed actively. Therefore, these changes must be recognized and managed to exploit the potential of distance monitoring services in improving healthcare demand management.

### Supporting mechanisms to manage the changes

**Fig. 3:**
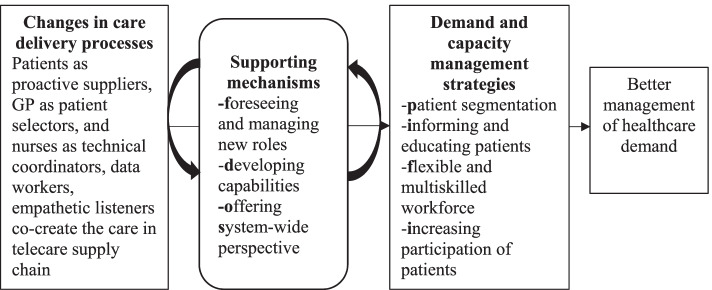
A framework to reduce healthcare demand using distance monitoring services

The findings suggest that, despite the potential of distance monitoring services, the fundamental challenge in managing healthcare demand remains the lack of adequate managerial provisions at the strategic level. To address this issue, we propose three supporting mechanisms: foreseeing and managing new roles, aligning capabilities with the potential of telemedicine, and adopting a system-wide perspective. We have updated the research framework (Fig. 3) with the findings and the mechanisms to present how healthcare demand can be better managed using distance monitoring services. In what follows, we elaborate on these mechanisms.

The emerging new roles should be acknowledged and formalized in telecare services. Although the literature has studied patients’ roles in care co-creation to an extent (e.g., [35]), we identify that deployment of distance monitoring significantly changes healthcare personnel’s roles as well. Management must recognize these changes in roles and related activities so that the actors can play these new roles with minimal challenges. Patients’ increased involvement in their treatment has been portrayed as empowering and positive development in the healthcare literature [76, 77]. An underlying assumption of this proposition could be that all patients are equally able and willing to take charge of their health using telemedicine. However, our study indicates otherwise. Patients were not always eager to use the app and upload their health data regularly, and GPs had to rigorously select patients who are suitable for the distance monitoring service. Informing and educating patients, which constitute a demand management strategy found in the service literature [50, 51], can be an important enabler under this circumstance. Letting the patients know about this service and what it requires from them could help them to make informed decisions about whether they want to receive this service or not. Moreover, the recognition of new roles played by GPs and nurses is the first necessary step in assessing whether and to what extent new skills are required to play these roles effectively. For example, nurses in distance monitoring services require a certain level of IT competence as technical coordinators and data workers [78]. Nurses, as empathetic listeners, communicate and provide care through the app instead of through an in-person interaction, requiring additional social and communication skills [79].

The current service configuration has lacked deliberate effort in developing such capabilities across the care delivery process. It was predicted that the competence required for digital health services will take time and grow incrementally [80]. The current on-the-job training, therefore, might not be sufficient for developing the capabilities required by these nurses. Concerning GPs’ roles as patient selectors, although professional knowledge and experience with patients provide initial support [81], a lack of guidelines for patient selection could lead to inconsistent service offerings and thus poor service quality [82, 83]. We posit that the data generated by telecare services could provide a scientific basis for creating these guidelines. This dataset includes patients’ longitudinal health data and also reveal their app use patterns. This rich dataset could be used to create simulation models to identify the profiles of suitable patients and the demand trends of such patients [84].

Finally, we argue that a system-wide perspective, instead of a provider or user perspective, is essential to implement distance monitoring services or any other telemedicine. Different organizations within the primary and specialized healthcare system are involved in the care delivery process, especially for patients with chronic and multimorbid conditions. Therefore, it is important to consider the system-wide impact of telemedicine implementation. Our study indicates that the distance monitoring service adds new activities for GPs and nurses and results in creating a response center as a new care delivery point. From the municipality perspective, the service increases the resource requirement. However, when seen from a system-wide perspective, the service can reduce the demand for specialized care and emergency care, in the end positively impacting healthcare demand management. A system-wide perspective also implies connecting operational and strategic activities. In this study, the identified changes are at the operational level of the service and are not planned but rather emerge as the consequences of telemedicine deployment. We argue that support mechanisms from the strategic level of service are required to manage these changes so that potential risks can be minimized and the effectiveness of telemedicine can be maximized.

### Research implications

Our research has valuable implications for both academia and practice. This study addresses the need for empirical research on healthcare demand management, which has been raised by many [20, 22, 85] and met by very few (e.g., [86, 87]). Questions have been raised about whether flexible and easier access to care, as offered by telecare services, and curbing demand for care can coexist [86]. This study offers an explanatory answer to these calls and questions by showing that services can better manage the healthcare demand while providing easy access to care. However, the deployment of technology might not be sufficient to achieve this balance. The implementation of the technology must be supported by these mechanisms to manage the changes triggered by the new technology.

Although studies focusing on digital health are rife, this study is among the few [81, 82] focusing on the effects of digitalization on collaboration and communication among different actors. Using the concept of co-creation and service triad, this study identifies that such services change the communication channel from face-to-face interaction to indirect, but more frequent, app-based interaction. Moreover, the need for collaboration among the main actors is increased for the service to function efficiently. The comprehensive identification of these changes also enables us to analyze the impact of such services on healthcare demand management. Both the research community and practitioners expect that telemedicine has a positive impact on demand and resource management in healthcare [49]. However, the current literature does not provide sufficient information on whether and how services such as distance monitoring can affect this issue. Our paper is one of the first to analyze the relationship between distance monitoring services and healthcare demand. In so doing, several aspects come into view that have not been discussed in the literature to date. The findings indicate that telemedicine can positively contribute to demand management, but it also adds complexities to the service, for example, making the service more dependent on patients’ proactive participation, requiring new skills, and adding new activities. These complexities, if not recognized and managed, can add uncertainties to the service and increase the use of resources, leading to reduced quality of care. The merit of such analysis is the comprehensive understanding of the managerial aspects of telemedicine that have been less investigated in the extant literature but are essential for better service configuration and improved care.

For practitioners and policy-makers, this study provides important insights related to new roles and competencies. The identified new roles and managerial mechanisms provide a means for managers to reflect on their planning and decision-making regarding telemedicine implementation. Often, healthcare organizations, as in our case, introduce telemedicine services hoping that they can reduce resource consumption. This study draws attention to telemedicine being able to facilitate certain strategies but managerial actions in designing telecare services remaining pivotal in addressing the escalating demand with limited resources. Finally, we draw policy-makers’ and practitioners’ attention to the need for new competencies and education and training to develop these competencies, which are essential for large-scale implementation, and use of telemedicine as a regular service as opposed to pilot projects or interventions.

### Limitations and direction for future research

Our study has a few limitations. Applying the co-creation and service triad concept was appropriate to comprehensively identify the changes in the care delivery process. However, it limits the use of management’s voice in our analysis since managers are not explicitly active in the care delivery process. Therefore, we used the data from management (Table 1) for triangulation purposes to corroborate the main data material. This paper focuses on telemedicine in a public healthcare setting. While the design enables in-depth analysis and better reflection, we believe that similar studies are needed in different contexts with similar and different IT provisions. For example, if a healthcare system decides that, instead of creating a response center, GPs will manage the distance monitoring app, how will this change affect co-creation processes and strategies, such as workforce flexibility and resource utilization? The findings of multiple studies could then be compared, and patterns of demand management could be cross-matched to devise precise theories on the relationship between telemedicine and healthcare demand management. Next, studies analyzing quantitative data on demand and resource utilization could advance the findings of this study by adding new empirical insights. Finally, our findings indicate that distance monitoring services provide new provisions for co-creating care by increasing patients’ active participation, which could be further investigated and analyzed in relation to patients’ quality of life in future studies.

## Conclusion

Meeting the healthcare demand with available resources is a global challenge. With the increasing size of the elderly population, chronic ailments, and multimorbidity, the disparity between demand and resources seems to deteriorate even more with time. Therefore, it is important to explore the merit of telemedicine in solving this pressing issue. Given that technology implementation in healthcare is a complex phenomenon, it is not sufficient to know only whether telemedicine can contribute to managing the escalating healthcare demand; we must also know how we can do so. Therefore, this paper first demonstrates the complex changes in the care delivery process triggered by the implementation of the distance monitoring service. Next, we explain the potential of the service to improve demand management by analyzing the fit between the service operations of the care delivery process and demand-capacity strategies from the literature. Our analyses also suggest that, in the absence of strategic support, the potential for telecare services to improve demand management cannot be exploited, and the service could have unintended consequences, such as added uncertainty and an increase in resource consumption. Therefore, we propose a set of managerial mechanisms for telemedicine services to bridge the gap between operations and strategic levels to improve healthcare demand management. This article contributes to the eHealth literature by identifying the specific potentials of distance monitoring services in improving demand management. It does so by demonstrating how the operational changes in terms of activities, interaction and roles pave the way for demand-capacity strategies to be implemented. The next contribution is to underscore the essential need for managerial mechanisms to connect the strategic level and operational level of eHealth services and specify those mechanisms. These contributions have serious implications for the large-scale implementation and sustainable use of telemedicine since the need for managerial readiness of implementing telemedicine is often overshadowed by the promises of the technical abilities of telemedicine.

## Supplementary Information

**Additional file 1.** Interview guide for municipality nurses.

**Additional file 2.** List of documents used as data sources as mentioned under the documents and archives section in the manuscript.

## Data Availability

The data collected and analyzed during the current study are not publicly available to maintain the confidentiality of the respondents, as well as the organizations and their processes, but they are available from the corresponding author upon reasonable request.
