# Supplementary material for: Impact of distance monitoring service in managing healthcare demand: a case study through the lens of cocreation
Source: BMC Health Serv Res. 2022 Jun 21;22:802. doi: 10.1186/s12913-022-08164-2 (PMC9209829; doi:10.1186/s12913-022-08164-2)
Supplement: Supplementary file 1 — Additional file 1. Interview guide for municipality nurses. [file 12913_2022_8164_MOESM1_ESM.docx]

# 1. Interview guide for municipality nurses

1. What position do you currently hold?
2. How long have you worked at the response centre?
3. What is your educational background?
4. Can you describe your job responsibilities?
5. How is a normal work-day for you? Can we go through the tasks that you do on a working day?
6. Can you compare your work before the distance monitoring service came and the way it is now?
7. How the tasks are allocated in the response center? How do you plan for work distribution and who does what?
8. Is your performance measured and assessed on a regular basis? If yes, can you explain the process?
9. Around how many patients do you talk to every day?
10. do you use audio or video call to talk to patients?
11. Besides patients, who else do you interact with for this job? (also ask how they carry out the interactions/communication, if not mentioned by the nurses)
12. Can you reflect on how your daily work has been changed due to the introduction of distance monitoring service?
13. Has anything changed in relation to the decisions you used to make and the decisions you make now?
14. Have you participated in some workshops or training sessions to learn how to use the app and related equipment?
15. How have you learned to use the app and related equipment?
16. Do you face any challenges in using those? If yes, how do you solve the issues?
17. Are there any challenges in working at the response center? If yes, what are they?

# 2. Interview guide for patients

1. Is it okay for you to share your diagnosis with me?
2. Do you remember how you heard about the distance monitoring service? (who told you, how…)
3. Since when are you using the distance monitoring app?
4. Do you use the app regularly?
5. How do you like the distance monitoring App?
6. What are the benefits of using the app in your view?
7. How does this new service affect the treatment you get?
8. Do you have any difficulties in using the App? If yes, how are these solved?
9. Have you ever talked to the nurse from the response center? (If yes, ask about the experience.)
10. Do you have any suggestion(s) for improving the distance monitoring service?

# 3. Interview guide for GPs

1. What position do you currently hold?
2. How long have you worked as a GP?
3. What are your job responsibilities?
4. Can you describe the tasks that you perform on a regular basis as a GP?
5. How did you come to know about distance monitoring services?
6. Could you explain what responsibilities you have in the distance monitoring service?
7. How does distance monitoring service help the patients?
8. Is there any other benefit of the service?
9. How do you present this service to the patients?
10. How do you decide on which patients to be offered the service?
11. What are the differences between the care delivery process of usual care and the one using distance monitoring app?
12. How are the municipality nurses involved in planning and delivering the care in distance monitoring service?
13. How are the various appointments, treatments, etc. planned for patients?
14. Which of these steps are affected by the distance monitoring app?
15. Can you elaborate on the types of patients (for example age, diagnosis type, location of residence) who are more eager to use distance monitoring service?
16. Can you explain the information flow within the care delivery process? (who are the actors you talk to and how what IT systems are in use)
17. How distance monitoring service changes the way of decision-making about treatment?
18. Do you think there are enough resources to deliver care properly in the current situation?
19. Do you think distance monitoring can help in better resource utilization and if yes, how can it do so?

# 4. Interview guide for Managers

1. What position do you currently hold?
2. For how long have you been working at X municipality?
3. What are your job responsibilities in general?
4. How would you describe the digitalization of healthcare and welfare technologies?
5. How do different ICT applications and digital platforms change the care delivery process in your opinion?
6. Can you elaborate on how the decisions are made in using different ICT tools/welfare technologies, for example, the process of selection, implementation, and involved actors?
7. What is the expectation from distance monitoring service? What value does it add to the Kommune?
8. What are the IT systems (for example patient journals at different care services) involved in the whole care delivery process?
9. How these systems are connected with each other?
10. Does the municipality use standardized patient care pathways (pasientforløp/pakkeforløp) for any group of patients?
11. What is your role and responsibilities in distance monitoring service?
12. Can you reflect upon the transition of this service from a project (HelseMi) to a regular service (Avstandsoppfølging)?
13. Can you describe the way(s) you communicate with different actors (for example, GP, nurses, administrators, specialized doctors, patients) involved in the care delivery process within this service?
14. Can you take me through the decision-making processes that you are part of within the service delivery processes?
15. Which of these decisions are made by a team and by an individual?
16. How distance monitoring service changes the way of decision-making about treatment?
17. Do you think there are enough resources to deliver care properly in the current situation?
18. Do you think distance monitoring can help in better resource utilization and if yes, how can it do so?
19. Do you think nurses have adequate skill and knowledge to use the app and equipment in distance monitoring services?
20. Do you think that the municipality provides enough training and support to learn about this app and equipment before starting working with it?

# 5. Interview guide for the distance monitoring app developer

1. Can you explain the distance monitoring app? (the following points need to be covered)
   1. What are the functionalities?
   2. How does it work?
   3. What patients can do with it and what do they get back from the app?
   4. What do the patients see in the app?
   5. What can nurses do with it and what do they get back from the app?
   6. What do the nurses see in the app?
   7. What are the changes that nurses can make?
   8. How does the data flow from the patients to the response centre?
2. Can you describe the development phases of this app?
3. Who were involved in the design of the app?
4. Who were involved in testing the app?
5. Can this app be modified for other diseases (other chronic diseases, mental health issues) that can be benefited from continuous monitoring and preventive support?
6. Do you have any plan to upgrade the app? If yes, how will the upgraded version be?
