# Supplementary material for: Impact of distance monitoring service in managing healthcare demand: a case study through the lens of cocreation
Source: BMC Health Serv Res. 2022 Jun 21;22:802. doi: 10.1186/s12913-022-08164-2 (PMC9209829; doi:10.1186/s12913-022-08164-2)
Supplement: Supplementary file 2 — Additional file 2. List of documents used as data sources as mentioned under the documents and archives section in the manuscript. [file 12913_2022_8164_MOESM2_ESM.docx]

| English title | Original title | Year of publication |
| --- | --- | --- |
| Effect of distance monitoring – a follow-up study | Effekter av avstandsoppfølging -følgeforskning av medisinsk avstandsoppfølging | 2018 |
| Distance monitoring of persons with chronic disease | Avstandsoppfølging av personer med kroniske sykdommer | 2018 |
| Recommendations for national implementation of distance monitoring service | Sluttanbefalinger fra utredningsoppdrag om  nasjonal tilrettelegging for medisinsk avstandsoppfølging | 2019 |
| Evaluation of distance monitoring trial- 1 | Evaluering av utprøving av digital hjemme-oppfølging - 1 | 2020 |
| My life, min health, my plan | Mitt liv, min helse, min plan | 2020 |
| Evaluation of distance monitoring trial- 2 | Evaluering av utprøving av digital hjemme-oppfølging - 2 | 2021 |

List of documents used as data sources as mentioned under the documents and archives section in the manuscript.
